# NHF-derived carbon dots: prevalidation approach in breast cancer treatment

**DOI:** 10.1038/s41598-020-69670-z

**Published:** 2020-07-29

**Authors:** Crina Elena Tiron, Gabriel Luta, Mihail Butura, Florin Zugun-Eloae, Corneliu S. Stan, Adina Coroaba, Elena-Laura Ursu, Gabriela Dumitrita Stanciu, Adrian Tiron

**Affiliations:** 1 Regional Institute of Oncology, TRANSCEND Center, 700483 Iasi, Romania; 2 Department of Immunology, “Gr.T.Popa” University of Medicine and Pharmacy, 700115 Iasi, Romania; 3 Department of Natural and Synthetic Polymers, “Gheorghe Asachi” Technical University of Iasi, 700050 Iasi, Romania; 4 Department of Chemistry, “Petru Poni” Institute of Macromolecular Chemistry, 700487 Iasi, Romania; 5 Center for Advanced Research and Development in Experimental Medicine (CEMEX), “Gr.T.Popa” University of Medicine and Pharmacy, Iasi, Romania

**Keywords:** Drug development, Preclinical research, Translational research, Cancer, Medical research, Oncology

## Abstract

Metastatic breast cancer dominates the female cancer-related mortality. Tumour-associated molecules represents a crucial for early disease detection and identification of novel therapeutic targets. Nanomaterial technologies provide promising novel approaches to disease diagnostics and therapeutics. In the present study we extend the investigations of antitumoral properties of Carbon Dots prepared from *N*-hydroxyphthalimide (CD-NHF) precursor. We evaluate the effect of CD-NHF on tumour cell migration and invasion in vitro and their impact on tumour progression using an in vivo model. Furthermore, we investigate the molecular mechanisms involved in CD-NHF antitumour effects. In vivo mammary tumours were induced in Balb/c female mice by injecting 4T1 cells into the mammary fat pad. Conditional treatment with CD-NHF significantly impair both migration and invasion of metastatic breast cancer cells. The presence of CD-NHF within the 3D cell cultures strongly inhibited the malignant phenotype of MDA-MB-231, 4T1 and MCF-7 cells in 3D culture, resulting in culture colonies lacking invasive projections and reduction of mammospheres formation. Importantly, breast tumour growth and metastasis dissemination was significantly reduced upon CD-NHF treatments in a syngeneic mouse model and is associated with down-regulation of Ki67 and HSP90 expression. CD-NHF nanostructures provide exciting perspective for improving treatment outcome in breast cancer.

## Introduction

In women, breast tumours still remain one of the leading causes of cancer-related deaths^1^. Currently, breast cancers are classified by different criteria including pathology (lobular, ductal), stage (TNM), grade, receptor status and presence or absence of gene mutation (e.g. BRACA1/2). Clinicopathological variables such as tumour size, tumour grade and nodal status together with immunohistochemistry (IHC) markers e.g. estrogen receptor (ER), progesterone receptor (PR) and human epidermal growth factor receptor 2 (HER2) are conventionally used for patient prognosis and therapeutic decision^2^. Therapeutic drugs like, tamoxifen (for ER positive tumours), trastuzumab (for HER2 overexpressing tumours) are used for treatment of distinct sub-types of breast cancer^3,4^. Inter- and intra-tumour heterogeneity have significant implications for breast cancer diagnosis and treatment efficiency^5^. However, drug resistance of cancer cells represent challenge for cancer therapy^6^. Targeted therapy for very aggressive sub-type like triple-negative breast cancer is still under investigation^7^.

The process by which cancer cells invade into surrounding tissues and migrate to distant organs, metastasis, is responsible for 90% of cancer related death. Metastasis is a cascade process characterized by detachment of tumour cells from the epithelial layer, incursion through the basement membrane into the adjacent tissue, intravasation, survival in the bloodstream, extravasation at a distant site and growth of secondary tumour at the target organ^8^. Epithelial to mesenchymal transition (EMT) plays a key role in the metastatic process. Many molecules involved in tumour progression are overexpressed in cancer cells which makes them useful biomarkers in cancer diagnostics. One of the most fundamental characteristics of cancer cells is the fast replication rate which provide an important information about a patient’s prognosis^9^. High level of the widely used proliferation marker, Ki67 is associated with poor disease-free survival, overall survival and breast cancer tumour recurrence^10^. Elevated levels of heat shock proteins (HSPs) has been observed in different types of human tumours, including breast, ovarian, endometrial, lung, colon, and prostate cancers playing key roles in carcinogenesis and tumour progression^11^. HSP90, a 90 kDa heat shock protein, is essential for maintaining functionality of important cellular proteins including protein kinases, protein involved in apoptosis and cell cycle^12^. It has been shown that heat shock proteins (HSP) are overexpressed in mammary carcinoma and high HSP90 expression was associated with decreased patient survival. Moreover, it has been reported that up-regulation of HSP90 expression is associated with an increased risk of recurrence of triple negative breast cancer^13^. The role of HSP expression in cancer development and its biological mechanisms are under investigation. It is clear that is necessary a novel therapeutic strategies to inhibit invasion, migration and metastasis.

Nano-technologies, has provided very exciting modalities for optimizing treatment outcomes in cancer and other types of disease. In this paper CD-NHF were prepared in a modified way of previously described method^14^ (e.g. 10 min pyrolysis instead of 20) and their effect was investigated in different cancer micro-environments. Since our previous results^15^ demonstrates down-regulation of vimentin, a marker of EMT that is involved in cell migration and invasion, we expanded our investigation of the mechanisms involved in the putative anti-metastatic effect of CD-NHF both in vitro and in vivo.

## Results

### CD-NHF significantly inhibits in vitro invasiveness, spheroid and mammospheres formation of breast cancer cells

**Figure 1:**
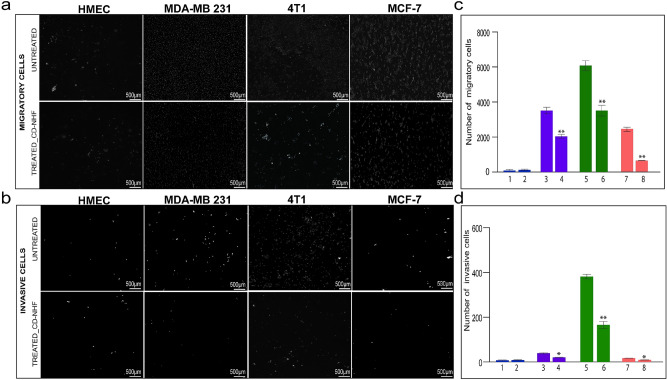
Effect of CD-NHF in Migration and Invasion assay. a. Migration; b. Invasion. **1,2** HMEC (1-untreated, 2-treated with 5% CD-NHF); **3,4** MDA-MB-231(3-untreated, 4-treated with 5% CD-NHF); **5,6** 4T1 (5-untreated, 6-treated with 5% CD-NHF); **7,8** MCF7 (7-untreated, 8-treated with 5% CD-NHF). N = 4 migration/invasion/experiment .*p < 0.05, **p < 0.005.

 Because breast cancer mortality is mainly related to complications of metastatic lesion, we assessed the effect of CD-NHF on breast cancer cell invasiveness. To effectively assess the role of CD-NHF in breast tumour progression we first investigated the influence of tested nanostructures in migration and invasion systems (Fig. 1). The directed movement of cells on a substrate is defined as migration^16^ while invasion of cancer cells is defined as penetration of the basement membrane and infiltration into underlying tissues which requires adhesion, proteolysis of extracellular matrix components and movement.

Analysis of breast carcinoma cell invasion through 3D (three-dimensional) extracellular matrix protein gels (Matrigel) shows a reduction of the ability of cancer cells to migrate and invade in the presence of 5% CD-NHF (Fig. 1a,b).

**Figure 2:**
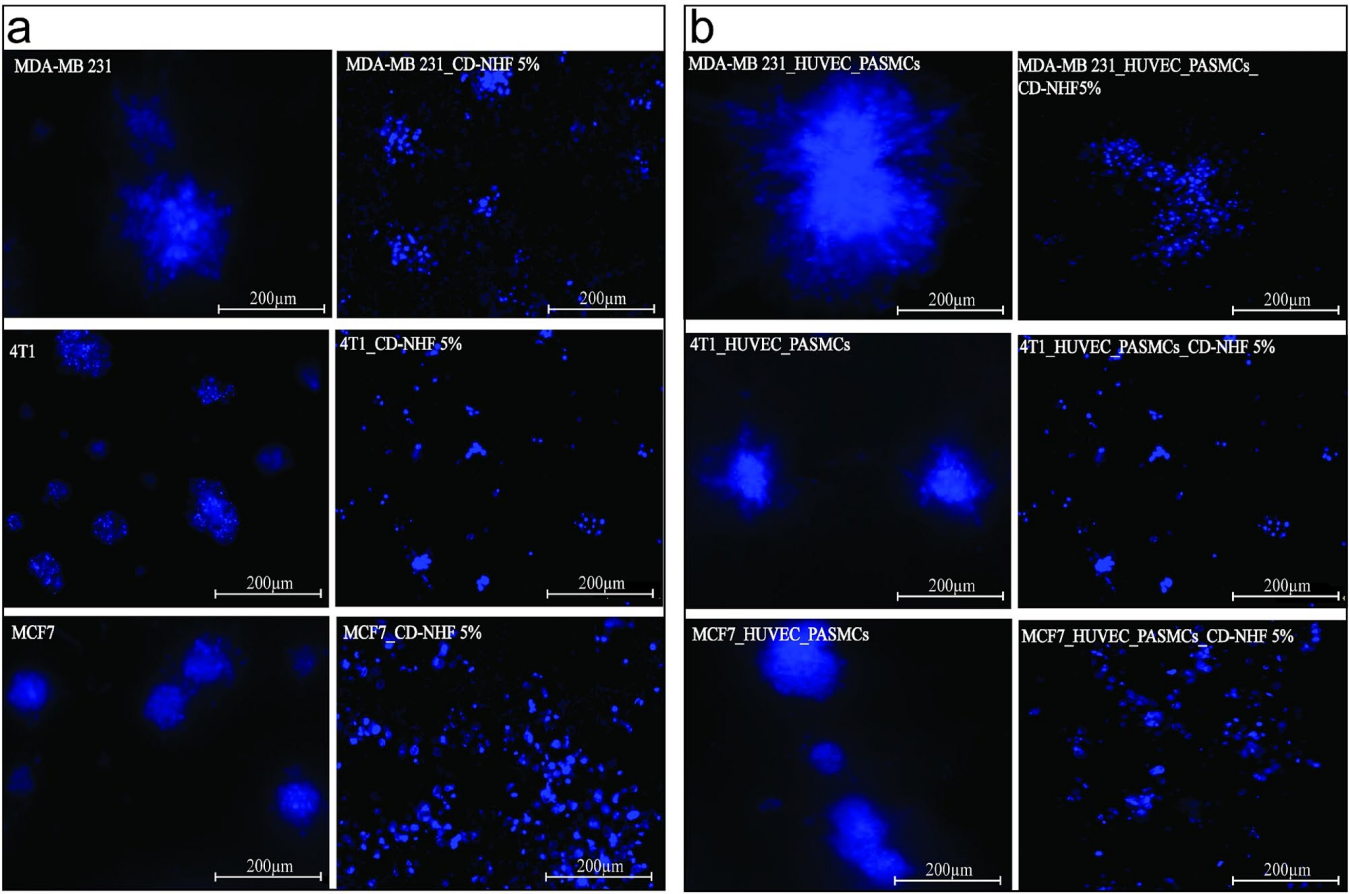
Effect of CD-NHF in 3D matrigel assay. **A** Monoculture.**a,b** MDA-MB-231 (a-untreated, b-treated); **c,d** 4T1 (c-untreated, d-treated); **e,f** MCF7 (e-untreated, f-treated); **B**. Co-culture **a,b** MDA-MB-231 (a-untreated, b-treated); **c,d** 4T1 (c-untreated, d-treated); **e,f** MCF7 (e-untreated, f-treated). 10×, N = 3 matrigels/experiment. Pictures acquired at 10×.

Cancer development is influenced by intra-tumour cellular interactions in which stromal cells support primary tumour growth and metastasis^17^. To explore whether CD-NHF influences the ability of malignant breast cancer cells to grow, we deployed a 3D three-cellular approach comprising breast cancer cells (MDA-MB231, 4T1 or MCF7) cells co-seeded with primary human vein endothelial cells (ECs) and vascular smooth muscle cells (vSMCs) into 3D matrigel in order to mimic tumours. Morphological aspect of 3D cell cultures right before applying CD-NHF treatment is represented in Figure S5. Normal breast epithelial cells are organized into spheroidal acinar structures in 3DMatrigel whereas malignant cells (e.g. MDA-MB-231, 4T1 and MCF7) in co-culture system formed larger colonies with stellated invasive cell projections that reflect aggressive tumours. These results are in line with migration and invasion capabilities, triple negative cell lines (MDA-MB-231 and 4T1) exhibiting an increased invasiveness compared to Her2 negative cell line (MCF7). Fluorescent analysis of cell cultures 10 days post CD-NHF treatments showed that the presence of CD-NHF strongly inhibited the malignant phenotype of breast cancer cells which generate small colonies with reduced invasive projections (Fig. 2).

Together, these findings indicate that CD-NHF are efficient in attenuating the mesenchymal-like invasiveness of metastatic breast carcinoma cells.

**Figure 3:**
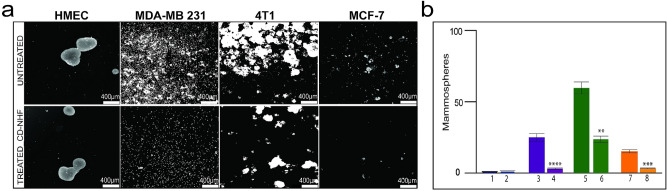
Effect of CD-NHF in Mammosphere assay. **A**. Morphologic aspects. **a,b** HMEC; **c,d** MDA-MB-231; **e,f** 4T1; **g,h** MCF7. **B**. Mammosphers quantification **1,2** Normal mammary cells (HMEC, Mcf-10a) (1-untreated, 2-treated with 5% CD-NHF); **3,4** MDA-MB231 (3-untreated, 4-treated with 5% CD-NHF); **5,6** 4T1 (5-untreated, 6-treated with 5% CD-NHF); **7,8** MCF7 (7-untreated, 8-treated with 5% CD-NHF ).10 ×, N = 3. Pictures acquired at 5 ×. mammospheres/experiment. **p < 0.005, ***p < 0.0005, ****p < 0.00005.

Breast cancer is originated and maintained by a small fraction of tumour initiating cells, called cancer stem cells (CSCs)^18^ that are responsible for tumour progression, metastasis, therapy resistance and tumour recurrence. The mammosphere assay is used to propagate mammary CSCs in vitro*.* The effect of CD-NHF in mammosphere formation was determined and CD-NHF significantly impair number of mammospheres formed by cancer cell lines while normal cell line mammospheres formations were not reduced (Fig. 3).

CD-NHF dramatically compromise efficiency of mammospheres formation and development in cancer cell line. Collectively, this findings indicate that the tested nanostructure impair invasiveness and breast cancer stem cell population.

### CD-NHF impair tumour growth and metastasis in the 4T1 in vivo breast carcinoma model

**Figure 4:**
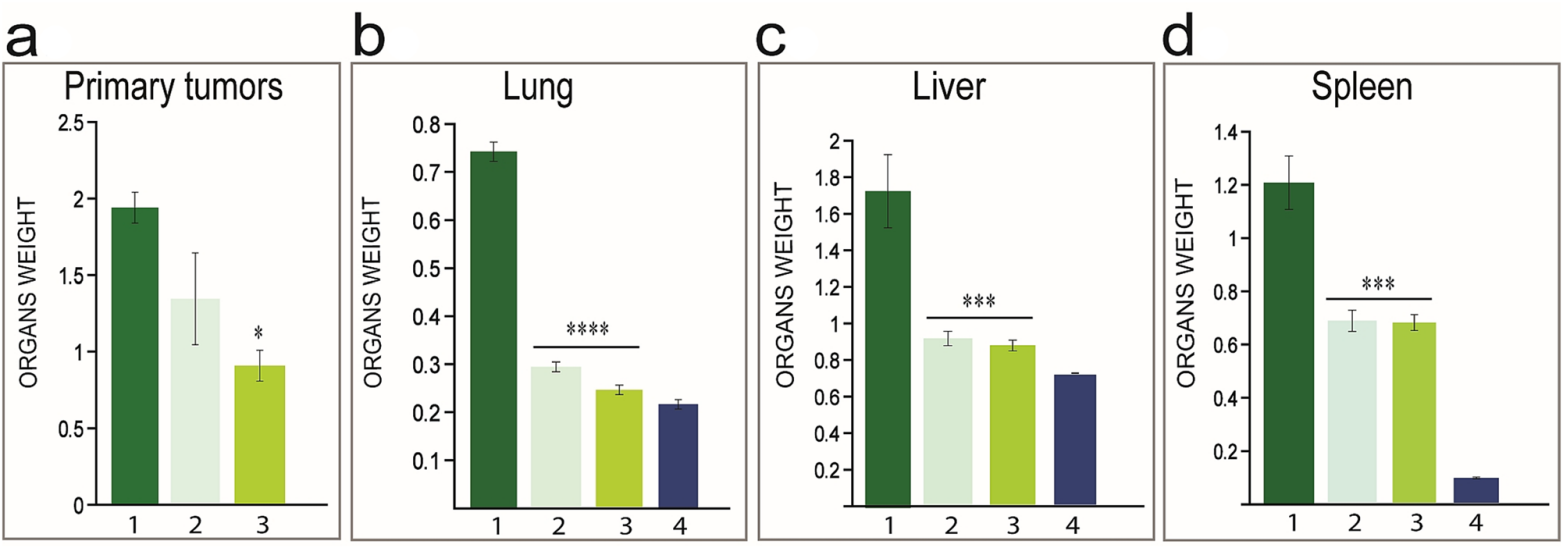
Organs weight. **A**. Primary tumours. **B**. Lungs. **C**. Liver **D**. Spleen. 1. tumour bearing mice untreated with CD-NHF; 2. tumour bearing mice treated with 10% CD-NHF; 3. tumour bearing mice treated with 20% CD-NHF; 4. Organs from tumour free mice . *p < 0.05, ***p < 0.0005,****p < 0.00005.

**Figure 5:**
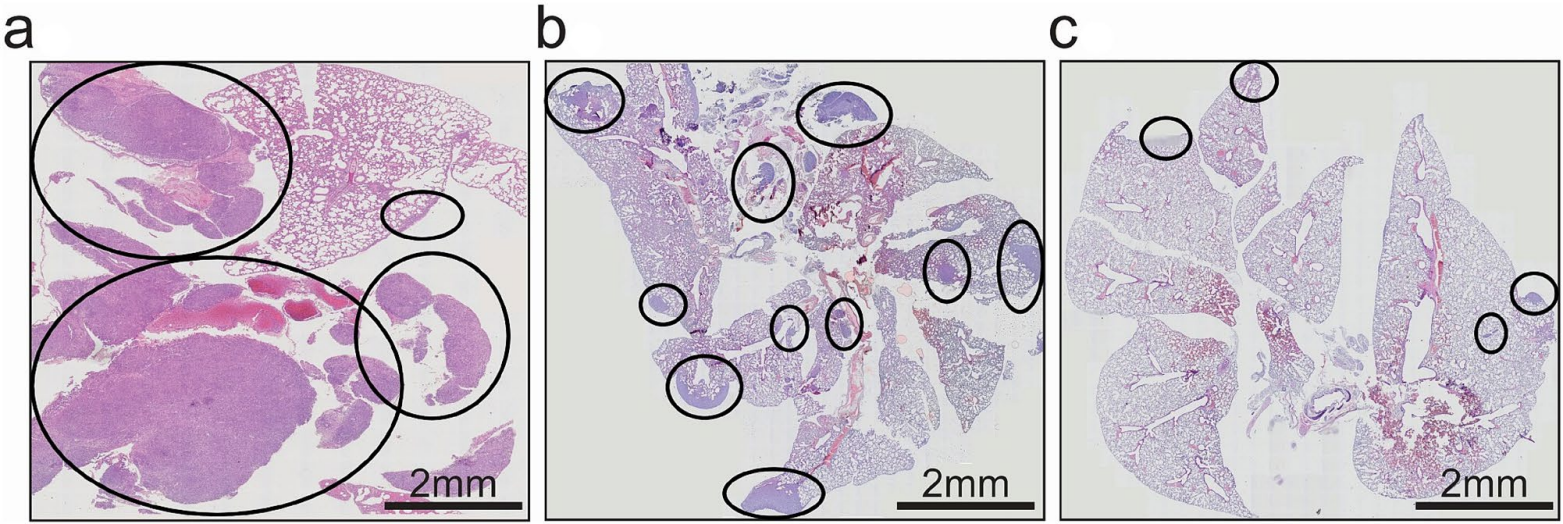
Microscopic aspects of lung metastasis. **a**. untreated **b**. treated 10% CD-NHF; **c**. treated 20% CD-NHF. Pictures acquired at 10×.

 To evaluate the influence of CD-NHF in tumour evolution and metastasis dissemination, we orthotopically inject 4T1 cells into mammary fat pad of Balb/c mice. The mouse mammary cancer cell line (4T1) is highly invasive and metastasize to lymph nodes, lungs, brain, liver, bone, ovary similar to triple negative stage IV breast cancer in women^19^. As we tested for the first time CD-NHF in vivo, it is difficult to translate working concentration from cell culture to experimental animal. We tested two concentration of CD-NHF for in vivo experiments, 10% and 20% relatively to mice blood volume, administered intraperitoneal twice per week. To avoid potential deleterious effects of high concentration CD-NHF administration we chose two concentrations as backup, if one is too small the other to may display a biological effect and if the highest is toxic for tested mice, the lower concentration will became working concentration. CD-NHF (dispersed in phosphate buffer saline) have been administered starting from 2 weeks post cancer cell inoculation. Treatment started at 2 weeks post tumour cells inoculation based on the facts that at this time point primary tumour was sufficiently developed and mice presents micro-metastases in distant organs. A smaller group of mice (N = 6) are tumour free and received 20% CD-NHF for the same period as treated group. The presence of metastases in organs is often reflected by increasing organs weight relatively to control ones. In our experimental setup 10% and 20% CD-NHF treatment reduced the rate of primary tumour growth and metastasis dissemination (Figs. 4 and 5).

After the animal were sacrificed the excised organs were prepared for further analysis. For histological analysis we focused on primary tumours and lungs. Lung represent primary metastatic target of 4T1 cell line and development of metastases in this organ is difficult to address. The histological analysis of tissue specimens showed the extensive metastasis in lungs from mice with 4T1 untreated tumours (Fig. 5a), while the mice treated with 10% and 20% CD-NHF showed reduction in lung metastasis formation (Fig. 5b,c).

**Figure 6:**
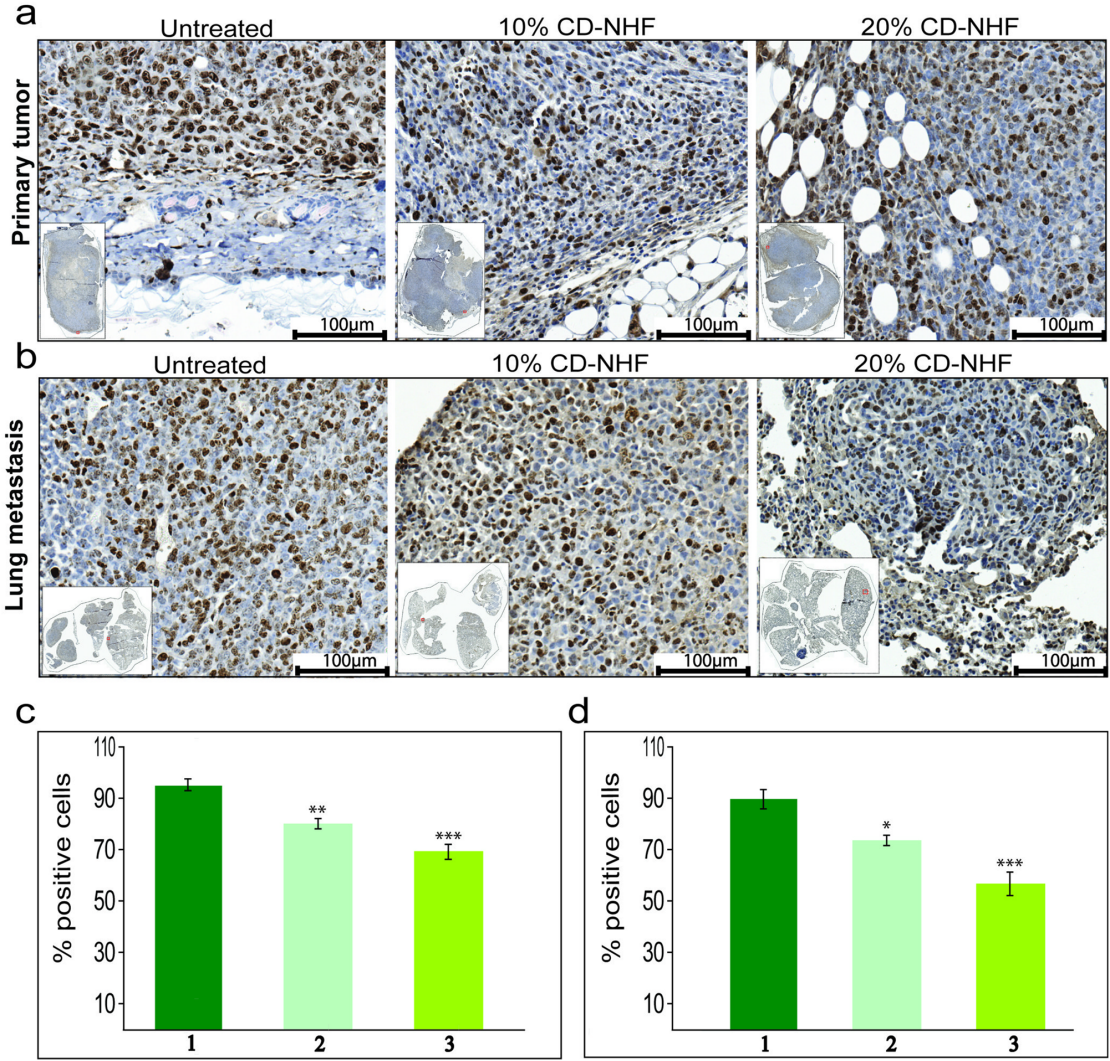
Ki67 expression. **A**. Primary tumours. 1. untreated; 2. treated with 10% CD-NHF; 3. treated with 20% CD-NHF. **B**. Lung metastasis. 1. untreated; 2. treated with 10% CD-NHF; 3. treated with 20% CD-NHF; **C**. Quantification of Ki67 level in Primary tumour; **D**. Quantification of Ki67 level in Lung metastasis. *p < 0.05,**p < 0.005, ***p < 0.0005. Pictures acquired at 20×.

These results support the conclusion that CD-NHF nanostructure impair in vivo breast tumour formation and metastasis spread. Uncontrolled proliferation represents an important features of malignancies and proliferative activity of tumour samples is used in order to determine the growth fraction of cancer cell populations. Solid tumours include a subset of cells, cancer stem cells (CSCs), that are characterized by their potential to self-renew and highest ability to grow in different in vivo tumour models^20^. Ki-67 depletion showed a reduction of this CSCs subpopulation, displayed reduced ability of tumour formation, suggesting that Ki-67 is required to maintain cancer stem cell niche^21^. In our work, at the end of experiment the expression of Ki67 was significantly reduced in both primary tumour and lung metastasis in 10% and 20% CD-NHF treated groups (Fig. 6).

**Figure 7:**
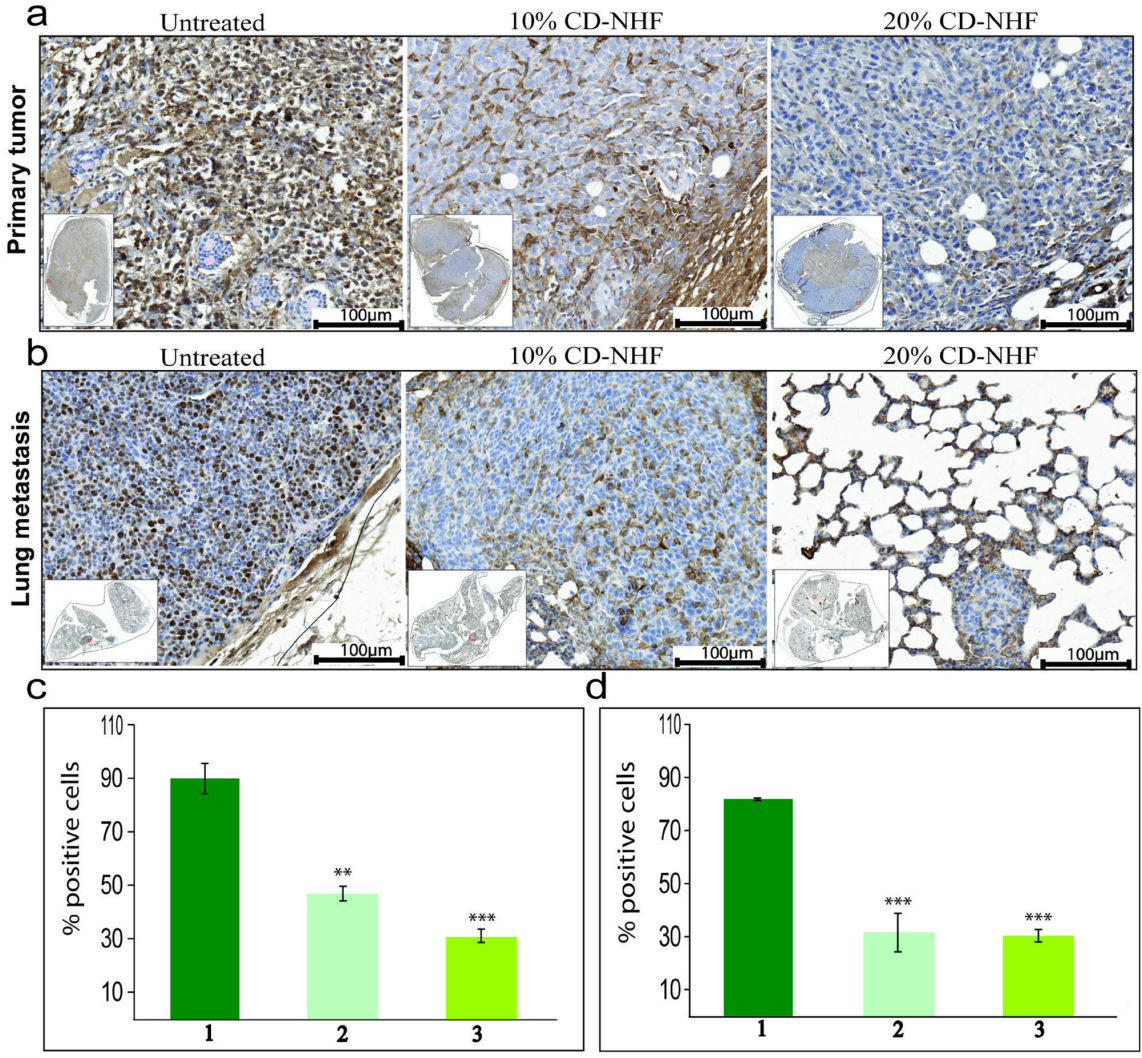
HSP90 expression. **A**. Primary tumours. 1. untreated; 2. treated with 10% CD-NHF; 3. treated with 20% CD-NHF. **B**. Lung metastasis. 1. untreated; 2. treated with 10% CD-NHF; 3. treated with 20% CD-NHF; **C**. Quantification of HSP level in Primary tumour; **D**. Quantification of HSP level in Lung metastasis. **p < 0.005, ***p < 0.0005 Pictures acquired at 20×.

In breast cancer, cell proliferation was correlated with up-regulation of stress-related genes HSPs. HSP90 play a important role in the multiple processes leading to tumour progression, invasion, metastasis and tumour immune response^22^. We investigate whether CD-NHF 10% and 20% may affect HSP90 expression in primary tumour and lung metastasis (Fig. 7).

Our results show that the level of HSP90 was affected, similar with Ki67, in a presence of tested nanostructures.

**Figure 8:**
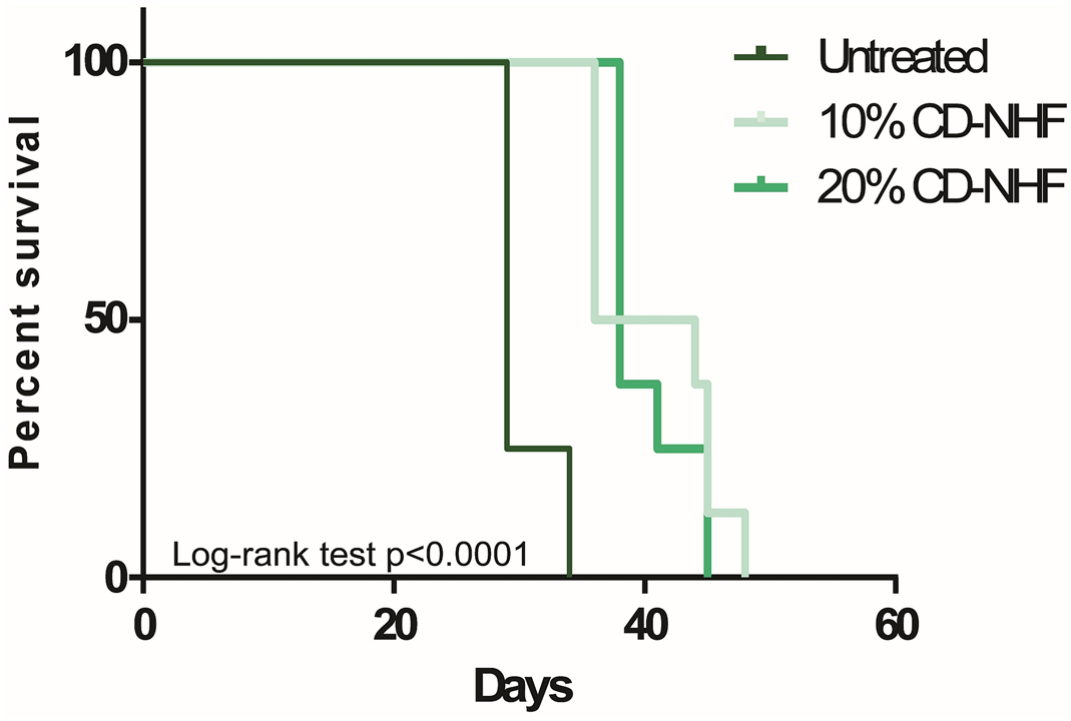
Univariate survival analysis between untreated and treated groups.

We evaluate also the contribution of CD-NHF treatments to cancer-related deaths (Fig. 8).

Overall late survival profile was significantly prolonged in both treated groups. Survival profile reflects primary tumour reduction and metastases impairment from collected organs. Although our data indicates that treatment with 20% CD-NHF exhibited a higher biological effects compared to 10%, survival data was similar for both concentrations, and, at this level of investigation, we think that dose increasing toxicity of CD-NHF might limit the overall beneficial effect.

## Discussion

We provide evidence that the CD-NHF treatment significantly inhibits breast cancer progression and metastasis both in vitro and in vivo. Our results suggest that tested nanostructure may interact in different molecular scenarios with various key molecules essential for invasiveness and spontaneous metastasis.

Nanomaterials have been stated to be one of the most promising platforms for cancer imaging and drug/gene delivery since they have the ability to combine diagnosis and therapy into one material^23–35^. We previously synthesized and characterised Carbon Dots from imide precursors and these Carbon Dots exhibited enhanced fluorescent emission relatively to other described Carbon Dots at that time^14^. In the present work, we extend our previous investigation on putative anti-tumoral properties of CD-NHF in both in vitro and in vivo systems. The structure of the precursor used to prepare the CD-NHF influences the biological response on different breast cell types. Our previous study^15^ demonstrated the down-regulation of vimentin upon CD-NHF treatment. Vimentin is a major indicator of cancer cell ability to migrate from primary tumor and invade to surrounding tissue. In the present study we did not investigated directly vimentin as a marker of EMT; we rather assessed migration and invasion processes which relay on partial or full EMT process which is clearly demonstrated to require vimentin regulation. Temporary increase of vimentin may not be always accompanied by a true invasion process, while migration and invasion is always accompanied by an increased vimentin expression. Migration and invasion, important steps in tumour progression was impaired by CD-NHF treatments with a remarkably effects in very aggressive sub-types like triple-negative breast cancer cell line (MDA-MB231 and 4T1). Meanwhile, treatments with similar dosage (5%, 50 µg/ml) do no have significant effects on normal cell types.

Tumour micro-environment is an important contributor in cancer development. Endothelial cells (ECs) cultured together with tumour cells improve tumour growth and blood vessel maturation was increased when co-seeded endothelial cells (ECs) and human pulmonary artery vascular smooth muscle cells(hPASMCs) compared with mono-cultures^36^. Blood vessels ensure proper oxygen and nutrient delivery to surrounding cells thus promoting tumour growth^37^. In order to investigate the ability of breast cancer cells treated with CD-NHF to co-opt/abrogate blood vessels we conducted three-cellular culture in 3D matrigel assay. Breast cancer cells co-seeded with endothelial cells (ECs) and smooth muscle cells (PaSMCs) form large organoids, while presence of 5% CD-NHF significantly reduced breast cancer organoids formation even in a presence of mimicking vasculature.

The presence of CSCs in variety of tumours are responsible for cancer progression, resistance to therapy and tumour recurrence and understanding their behaviour is an important step in treatments development^38^. Our results indicate that 5% CD-NHF weakened stem-like self-renewal properties evaluated by mammosphere assay.

Triple-negative breast cancer, the most aggressive breast cancer sub-type do to high incidence of disease-recurrence, highly metastatic potential, less options of tumour-specific treatments is currently the focus of intensive research^39^. Murine breast tumour 4T1 is an extremely metastatic syngeneic BALB/c model representing the triple-negative breast cancer subtype with very similar pattern of human disease. However, we observed that orthotopic breast tumour formation within the mammary gland was significantly reduced upon 20% CD-NHF treatments, while 10% CD-NHF do not affect primary tumour formation. Furthermore, we found significantly larger sizes and weights of organs in untreated mice compare with treated specimens. Histological analysis on different organs revealed significantly less metastasis in mice treated with 10% or 20% CD-NHF. The reduction of spleen weight under both doses of CD-NHF, when referred to no treatment, appears to be less impressive when compared to lung and liver. In the case of the spleen this result might be due to its plasticity, involvement in immunologic events and the fact that in this type of breast cancer there is an extensive extramedullary hematopoiesis. All these biological effects of CD-NHF are further sustained by the expressions of proliferation marker Ki67 and stress-related genes HSP90 which were significantly reduced in both primary tumour and lung metastasis upon both CD-NHF treatments. In vitro reduction of cancer stem cells upon CD-NHF treatment and the impaired cancer cells viability, migration, invasion and metabolism^40^ are linked to reduction of proliferation marker Ki67 in in vivo model. Our survival data of tested mice reflects cancer development impairment under CD-NHF treatments and the fact that 20% CD-NHF exhibited an increasing toxicity compared to 10% CD-NHF which limit the overall beneficial effect. It has been reported that nanostructures may induce endothelial leakiness which in turn promote tumour cell dissemination that could explain the similar late survival rate in both treatments.

At this moment, we do not know the exact mechanisms of the anti-tumour effect exhibited by CD-NHF. We have tested concentrations ranging from 0.1 to 10% CD-NHF on normal vs tumoral cell lines. The anti-tumor effect of CD-NHF on cancer cells viability impact without to impair normal cells viability was maximum at 5%; the10% concentration significantly reduced also normal cells viability. We believe that, despite many common and different cell processes occur in cancer vs normal cells, do to their size and tested concentrations, CD-NHF interfere mainly with metabolism of cancer cell which is different from normal cell. At higher concentrations (more than the10% we have tested), CD-NHF may mechanically block (sequestrate) some receptors by forming clusters due to their agglomeration tendency at higher concentrations, but in our working concentrations we can exclude this mechanism.

Collectively, this data demonstrate the therapeutic role of CD-NHF in both in vitro and in vivo models. The anti-tumoral therapeutic role may be further improved by combining reduced CD-NHF concentrations with other anti-cancer agents currently used in clinic. To extend the carbon dots investigations and their imaging and therapeutic applications, we developed carbon dots complexes^41^, that in addition to their anti-tumoural properties, are intended to be used for imaging via MRI.

## Materials and methods

### In vitro systems

*CD-NHF synthesis and characterisation* CD-NHF were synthesised form N-Hydroxyphthalimide (NHF) ( Sigma-Aldrich, 97%) using high purity water for preparation, purification and re-dispersion. CD-NHF were prepared through controlled pyrolysis of NHF for 10 min at 210^o^ C using a modified experimental protocol of the previously detailed protocol^14^. Briefly, the process undergoes under N_2_ atmosphere, the final decomposition product being suddenly flooded with cooled (4–5 °C) high purity water. The dispersion is centrifuged at 15,000 RPM for 10 min followed by the collection of the supernatant which is further centrifuged again in same conditions. The obtained aqueous dispersion containing purified and dimensionally selected CD-NHF are freeze dried and re-dispersed in water or other solvents according to application requirements. After synthesis, CD-NHF have been physico-chemical characterized.

*XPS analysis* was performed on a KRATOS Axis Nova, using AlKa radiation with a 20 mA current and 15 kV voltage with the incident X-ray beam focused on a 0.7 mm × 0.3 mm area. The wide XPS spectrum was collected within -10 to 1,200 eV range with 1 eV resolution and a pass energy of 160 eV. The high resolution spectra were collected using a pass energy of 20 eV and a step size of 0.1 eV (S1, S2 Fig). S1 and S2 Table contain detailed atomic and mass concentrations of the contained elements.

*Dimensional analysis* was performed on a Shimadzu SALD-7002 equipment. The size of the CD-NHF is ranging between 15–50 nm with an average size located within 20–30 nm interval (S3 Fig).

*AFM investigation* was performed using an Ntegra Spectra (NT-MDT, Russia) instrument provided with silicon cantilever tips (NSG 10) operated in tapping mode (S4 Fig). The recorded images reveal the tendency of agglomeration with clusters in 50–150 nm range which are evenly spread on the mica substrate.

*Cell cultures* All cells were cultured in a humidified atmosphere at 37 °C with 5% CO2. Human umbilical vein endothelial cells (HUVECs) (Lonza) and pulmonary artery smooth muscle cells (PASMCs) (Cambrex) were maintained in culture in the supplier's recommended complete medium (EGM-2 or SMGM-2; Lonza). The maximum passage number of cells used for experiments was 9 for HUVECs and 10 for SMCs. The human mammary epithelial cell line HMEC (Lonza) was cultured in MEBM/MEGM bullet kit. MCF-7 (ATCC) human breast adenocarcinoma cell line were cultured in Minimum Essential Medium (Cat. No. 30–2003) supplemented with the following components: 0.01 mg/ml human recombinant insulin; fetal bovine serum to a final concentration of 10%. 4T1 mouse breast carcinoma cells (ATCC, CRL-2539) and MDA-MB-231 human breast epithelial carcinoma cells (ATCC) were cultured as described by Gjerdrum et al.^37^.

*Invasion assay and migration assay* The assays was carried out using Cytoselect 24-well cell migration and invasion assay according to manufacture instructions (Cell Biolabs, INC CBA-100-C). Cells were seeded at 5 × 10^5^ cells per well in serum-free medium supplemented with 0.1% BSA. The cells were induced to migrate or invade toward medium containing 10% FBS alone or with 5% CD-NHF (5%, 50 µg/ml) for 24 h in the CO2 incubator. Groups treated with CD-NHF contained 5% CD-NHF also in the insert medium. 24 h post seeding non-invading cells were removed with a cotton swab. The remaining cells were fixed, stained with DAPI, and analysed by fluorescence microscopy (4 × magnification; Zeiss Axio Observer Z.1 microscope, TissueGnostics rig). Three images were acquired per well using TissueFAXS 4.2 software (provided by TissueGnostics) and quantified by using ImageJ software (ImageJ bundled with 64-bit Java 1.8.0_112, https://imagej.nih.gov/ij/download.html)..

*Co-culture assay* ECs and PaSMCs and tumour cells were mixed and seeded in culture plates in a 2:2:1 ratio in EGM-2 medium (Lonza) with or without CD-NHF. Networks were imaged by fluorescence microscopy after 10 days posttreatment, nuclei being stained with DAPI.

*Mammospheres assay* Mammospheres cultures of HMEC, MDA-MB-231, 4T1 and MCF7 cell lines were performed as previously described by Dontu et al.^42^.

## In vivo tumour model

*Mouse strain and animal care* The experiments were approved by Ethical Committee of the "Grigore T. Popa'' University of Medicine and Pharmacy of Iasi and were performed in accordance with the European legislation on the protection of animals used for scientific purpose and with authorization from National Sanitary Veterinary and Food Safety Authority (no. 17/02.07.2019). Female BALB/c mice (8–10 weeks old; Cantacuzino Institute, Bucharest, Romania) were used. The mice were housed in the animal facility of the CEMEX, ‘‘Grigore T. Popa’’ University of Medicine and Pharmacy, Iasi; in individually ventilated cages (IVCs) in a climate-controlled: 20 ± 4° Celsius, 50 ± 5% relative humidity and 12 h light/dark cycles; containing shaving bedding material, with regular rodent chow and water ad libitum.

*Mammary fat pad spontaneous metastasis model* 4T1 mouse breast carcinoma cells were suspended in MEM-EBSS medium/Matrigel (1:1) (1 × 10^6^ in 50 μL) and injected into the mammary pad of female BALB/c mice under depth anaesthesia. tumour growth was monitored two times/week and noted in laboratory book. At 2 weeks post tumour cells inoculation mice has been started to be treated via intraperitoneal injection with 10% (N = 9) or 20% (N = 9) CD-NHF for 4 weeks. CD-NHF concentration is relative to mice blood volume.

*Tissue collection* Mice were sacrificed 6 weeks after tumour cell inoculation by neck dislocation under deep anaesthesia. The primary tumours and different organs were retrieved from the mice and preserved in 10% paraformaldehyde (Sigma-Aldrich) for further analysis ( histopathologically by haematoxylin-eozin staining and by immunohistochemistry).

*Immunohistochemistry (IHC)* IHC was performed on 4 µm thick sections of formalin fixed and paraffin embedded tissues. Antigen retrieval was performed using Dako PT Link (pretreatment module) for 30 min in Target Retrieval Solution buffer (pH6 Dako K8005) (1:10 dilution in MilliQ). The slides were incubated overnight at 4 °C with antibody against Ki67 (Santa Cruz Biotechnology, 23,900) and HSP90 (Cell Signalling Technology, 4,875). Immunoperoxidase staining was carried out using the Dako Envision Flex kit (K8023) with DAB (diaminobenzidin tetrachloride) peroxidase as a substrate before counter-staining with Hematoxylin (ThermoScientific, 7,211). TissueFAXS 4.2 software (provided by TissueGnostics) has been used to acquire serial pictures and rebuild stained sections in digital format. Analysis for investigated markers expression has been done using HistoQuest 6.0 software (provided by TissueGnostics). In primary tumours markers were investigated at the invasion sites.

*Statistics* GraphPad Prism 6 for Windows was used for statistical analysis. Grouped analyses were performed by one-way ANOVA. Significance was established for p < 0.05.

## Supplementary information

Supplementary information

## References

[1] Siegel, R. L., Miller, K. D. & Jemal, A. Cancer Statistics. *CA Cancer J Clin.* **68**(1), 7–30. 10.3322/caac.21442 (2018). 29313949 10.3322/caac.21442

[2] Blows, F. M. *et al.* Subtyping of breast cancer by immunohistochemistry to investigate a relationship between subtype and short and long term survival: a collaborative analysis of data for 10,159 cases from 12 studies. *PLoS Med.* **7**, e1000279 (2010). 20520800 10.1371/journal.pmed.1000279 PMC2876119

[3] Brauch, H. & Schwab, M. Prediction of tamoxifen outcome by genetic variation of CYP2D6 in post-menopausal women with early breast cancer. *Br. J. Clin. Pharmacol.* **77**, 695–703 (2014). 24033728 10.1111/bcp.12229 PMC3971985

[4] Heyde, S. *et al.* mRNA profiling reveals determinants of trastuzumab efficiency in HER2-positive breast cancer. *PLoS ONE* **10**, e0117818 (2015). 25710561 10.1371/journal.pone.0117818 PMC4339844

[5] Marusyk, A., Almendro, V. & Polyak, K. Intra-tumour heterogeneity: a looking glass for cancer?. *Nat. Rev. Cancer* **12**(5), 323–334. 10.1038/nrc3261 (2012). 22513401 10.1038/nrc3261

[6] Reinhardt, F. *et al.* Navigation through inter- and intratumoural heterogeneity of endocrine resistance mechanisms in breast cancer: a potential role for liquid biopsies?. *Tumour Biol.* **11**, 1010428317731511. 10.1177/1010428317731511 (2017). 10.1177/1010428317731511 29129161

[7] Liu, D. *et al.* The smart drug delivery system and its clinical potential. *Theranostics* **6**(9), 1306–1323 (2016). 27375781 10.7150/thno.14858 PMC4924501

[8] Chambers, A. F. *et al.* Dissemination and growth of cancer cells in metastatic sites. *Nat. Rev. Cancer* **2**(8), 563–572 (2002). 12154349 10.1038/nrc865

[9] Hanahan, D. & Weinberg, R. A. Hallmarks of cancer: the next generation. *Cell* **144**(5), 646–674. 10.1016/j.cell.2011.02.013 (2011). 21376230 10.1016/j.cell.2011.02.013

[10] Inwald, E. C. *et al.* Ki-67 is a prognostic parameter in breast cancer patients: results of a large population-based cohort of a cancer registry. *Breast Cancer Res. Treat.* **139**(2), 539–552. 10.1007/s10549-013-2560-8 (2013). 23674192 10.1007/s10549-013-2560-8 PMC3669503

[11] Ciocca, D. R. & Calderwood, S. K. Heat shock proteins in cancer: diagnostic, prognostic, predictive, and treatment implications. *Cell Stress Chaperones* **10**, 86. 10.1379/CSC-99r.1 (2005). 16038406 10.1379/CSC-99r.1 PMC1176476

[12] Wegele, H. *et al.* Hsp70 and Hsp90—a relay team for protein folding. *Rev. Physiol. Biochem. Pharmacol.* **151**, 1–44. 10.1007/s10254-003-0021-1 (2004). 14740253 10.1007/s10254-003-0021-1

[13] Pick, E. *et al.* High HSP90 expression is associated with decreased survival in breast cancer. *Cancer Res.* **67**, 2932–2937 (2007). 17409397 10.1158/0008-5472.CAN-06-4511

[14] Stan, C. S. *et al.* Facile preparation of highly luminescent composites by polymer embedding of carbon dots derived from N-hydroxyphthalimide. *J. Mater. Sci.* **52**(1), 185–196. 10.1007/s10853-016-0320-y (2017).

[15] Tiron, C. E. *et al.*. Imide derived carbon dots exhibit promising antitumoral properties on multiple *In vitro* experimental designs. *Nano Tech. Nano Sci .Ind .J.* **13**(2):131.

[16] Friedl, P. & Wolf, K. Plasticity of cell migration: a multiscale tuning model. *J. Cell Biol.* **188**, 11–19 (2010). 19951899 10.1083/jcb.200909003 PMC2812848

[17] Montel, V. *et al.* tumour stromal interaction reciprocally modulate gene expression patterns during carcino-genesis and metastasis. *Int. J. Cancer* **119**, 25163 (2006). 10.1002/ijc.21757 16482564

[18] Schulenburg, A. *et al.* Neoplastic stem cells: a novel therapeutic target in clinical oncology. *Cancer* **107**, 2512–2520 (2006). 17039500 10.1002/cncr.22277

[19] Pulaski, B. A., & Ostrand-Rosenberg, S. Mouse 4T1 breast tumour model. *Curr .Protocol Immunol* 20.2.120.2.16 (2000). 10.1002/0471142735.im2002s39 18432775

[20] Al-hajj, M. *et al.* Prospective identification of tumourigenic breast cancer cells. *Proc. Natl. Acad. Sci. USA* **100**(7), 3983–3988 (2003). 12629218 10.1073/pnas.0530291100 PMC153034

[21] Cidado, J. *et al.* Ki-67 is required for maintenance of cancer stem cells but not cell proliferation. *Oncotarget* **7**, 6281–6293 (2016). 26823390 10.18632/oncotarget.7057 PMC4868756

[22] Bagatell, R. & Whitesell, L. Altered HSP90 Function in Cancer: A Unique Therapeutic Opportunity. *Mol. Cancer Ther.* **3**(8), 1021–1030 (2004). 15299085

[23] Chen, D. *et al.* Theranostic applications of carbon nanomaterials in cancer: Focus on imaging and cargo delivery. *J. Control. Release*. 10.1016/j.jconrel.2015.04.0211 (2015). 10.1016/j.jconrel.2015.04.021 25910580

[24] Núñez, C., et al. Novel Quinoline Molecular Probe and the Derived Functionalized Gold Nanoparticles: Sensing Properties and Cytotoxicity Studies in MCF-7 Human Breast Cancer Cells 10.1016/j.jinorgbio.2014.04.007. 10.1016/j.jinorgbio.2014.04.007 24861645

[25] Zhang, J. *et al.* Doxorubicin-Loaded Carbon Dots Lipid-Coated Calcium Phosphate Nanoparticles for Visual Targeted Delivery and Therapy of tumour. *Int. J. Nanomed.* **15**, 433–444 (2020). 10.2147/IJN.S229154 PMC6982446 32021189

[26] Kong, T. *et al.* Doxorubicin conjugated carbon dots as a drug delivery system for human breast cancer therapy. *Cell Prolif.* **51**, e12488 (2018). 30039515 10.1111/cpr.12488 PMC6528846

[27] Baker, S. N. & Baker, G. A. Luminescent carbon nanodots: emergent nanolights angew. *Chem. Int. Ed.* **49**, 6726–6744 (2010). 10.1002/anie.200906623 20687055

[28] Hola, K., et al. Carbon dots—–Emerging light emitters for bioimaging, cancer therapy and optoelectronics. Nano Today, 2014, 590–603. 10.1016/j.nantod.2014.09.004

[29] Mohammadia, S. *et al.* A FRET immunosensor for sensitive detection of CA 15–3 tumour marker in human serum sample and breast cancer cells using antibody functionalized luminescent carbon-dots and AuNPs-dendrimer aptamer as donor-acceptor pair. *Anal. Biochem.* **557**, 18–26 (2018). 29908158 10.1016/j.ab.2018.06.008

[30] Pardo, J., Peng, Z. & Leblanc, R. M. Cancer targeting and drug delivery using carbon-based quantum dots and nanotubes. *Molecules* **23**, 378 (2018). 29439409 10.3390/molecules23020378 PMC6017112

[31] Wang, H. Bi, J., Zhu, B. W. & Tan, M. Multicolorful carbon dots for tumor theranostics. *Curr Med Chem.* **25**(25), 2894–2909. 10.2174/0929867324666170316110810 (2018). 10.2174/0929867324666170316110810 28302015

[32] Zeng, Q. *et al.* Carbon dots as a trackable drug delivery carrier for localized cancer therapy in vivo. *J. Mater. Chem. B* **4**, 5119–5126 (2016). 32263509 10.1039/c6tb01259k

[33] Lin, X. J. *et al.* Multifunctional fluorescent carbon dots inhibit the invasiveness of lung cancer cells. *New J. Chem.* **42**, 15311–15314 (2018).

[34] Feng, T., Ai, X., An, G., Yang, P. & Zhao, Y. Charge-convertible carbon dots for imaging-guided drug delivery with enhanced in vivo cancer therapeutic efficiency. *ACS Nano* **10**(4), 4410–4420 (2016). 26997431 10.1021/acsnano.6b00043

[35] Gaddam, R. R., Mukherjee, S., Punugupati, N., Vasudevan, D. & Kothapalli, R. Facile synthesis of carbon dot and residual carbon nanobeads: Implications for ion sensing, medicinal and biological applications. *Materi. Sci. Eng. C* **73**, 643–652. 10.1016/j.msec.2016.12.095 28183656

[36] Carmeliet, P. & Jain, R. K. Molecular mechanisms and clinical applications of angiogenesis. *Nature* **473**(7347), 298–307. 10.1038/nature10144 (2011). 21593862 10.1038/nature10144 PMC4049445

[37] Gjerdrum, C. *et al.* Axl is an essential epithelial-to-mesenchymal transition-induced regulator of breast cancer metastasis and patient survival. *Proc. Natl. Acad. Sci. USA* **107**(3), 1124–1129 (2010). 20080645 10.1073/pnas.0909333107 PMC2824310

[38] Fillmore, C. M. & Kuperwasser, C. Human breast cancer cell lines contain stem-like cells that self-renew, give rise to phenotypically diverse progeny and survive chemotherapy. *Breast Cancer Res.* **10**, R25 (2008). 18366788 10.1186/bcr1982 PMC2397524

[39] Zhang, J. *et al.* Chemotherapy of metastatic triple negative breast cancer: experience of using platinum-based chemotherapy. *Oncotarget* **6**, 43135–43143 (2015). 26447756 10.18632/oncotarget.5654 PMC4767497

[40] Savin, C.-L. *et al.* Entrapment of N-Hydroxyphthalimide Carbon Dots in Different Topical Gel Formulations: New Composites with Anticancer Activity. *Pharmaceutics* **11**, 303. 10.3390/pharmaceutics11070303 (2019). 31266139 10.3390/pharmaceutics11070303 PMC6680570

[41] Stan, C. S., Coroaba, A., Ursu, E. L., Secula., M. S. & Siminonescu, B. C. Fe(III) doped carbon nanodots with intense green photoluminescence and dispersion medium dependent emission. *Sci. Rep.*. 10.1038/s41598-019-55264-x (2019). 10.1038/s41598-019-55264-x PMC6906313 31827161

[42] Dontu, G. *et al.* In vitro propagation and transcriptional profiling of human mammary stem/progenitor cells. *Genes Dev.* **17**(10), 1253–1270 (2003). 12756227 10.1101/gad.1061803 PMC196056

