# Correction to: NHF-derived carbon dots: prevalidation approach in breast cancer treatment

**DOI:** 10.1038/s41598-025-07302-0

**Published:** 2025-07-21

**Authors:** Crina Elena Tiron, Gabriel Luta, Mihail Butura, Florin Zugun‑Eloae, Corneliu S. Stan, Adina Coroaba, Elena‑Laura Ursu, Gabriela Dumitrita Stanciu, Adrian Tiron

**Affiliations:** 1 Regional Institute of Oncology, TRANSCEND Center, 700483 Iasi, Romania; 2 Department of Immunology, “Gr.T.Popa” University of Medicine and Pharmacy, 700115 Iasi, Romania; 3 Department of Natural and Synthetic Polymers, Gheorghe Asachi” Technical University of Lasi, 700050 Iasi, Romania; 4 Department of Chemistry, “Petru Poni” Institute of Macromolecular Chemistry, 700487 Iasi, Romania; 5 Center for Advanced Research and Development in Experimental Medicine (CEMEX), “Gr.T.Popa” University of Medicine and Pharmacy, Iasi, Romania

Correction to: *Scientific Reports* 10.1038/s41598-020-69670-z, published online 29 July 2020

The original Article contains an error in Figure 2. During the preparation of Figure 2b the image for the condition “4T1_HUVEC_PASMCs_CD-NHF 5%” was erroneously duplicated from the condition “4T1_CD-NHF-5%” in Figure 2a.

**Fig. 2:**
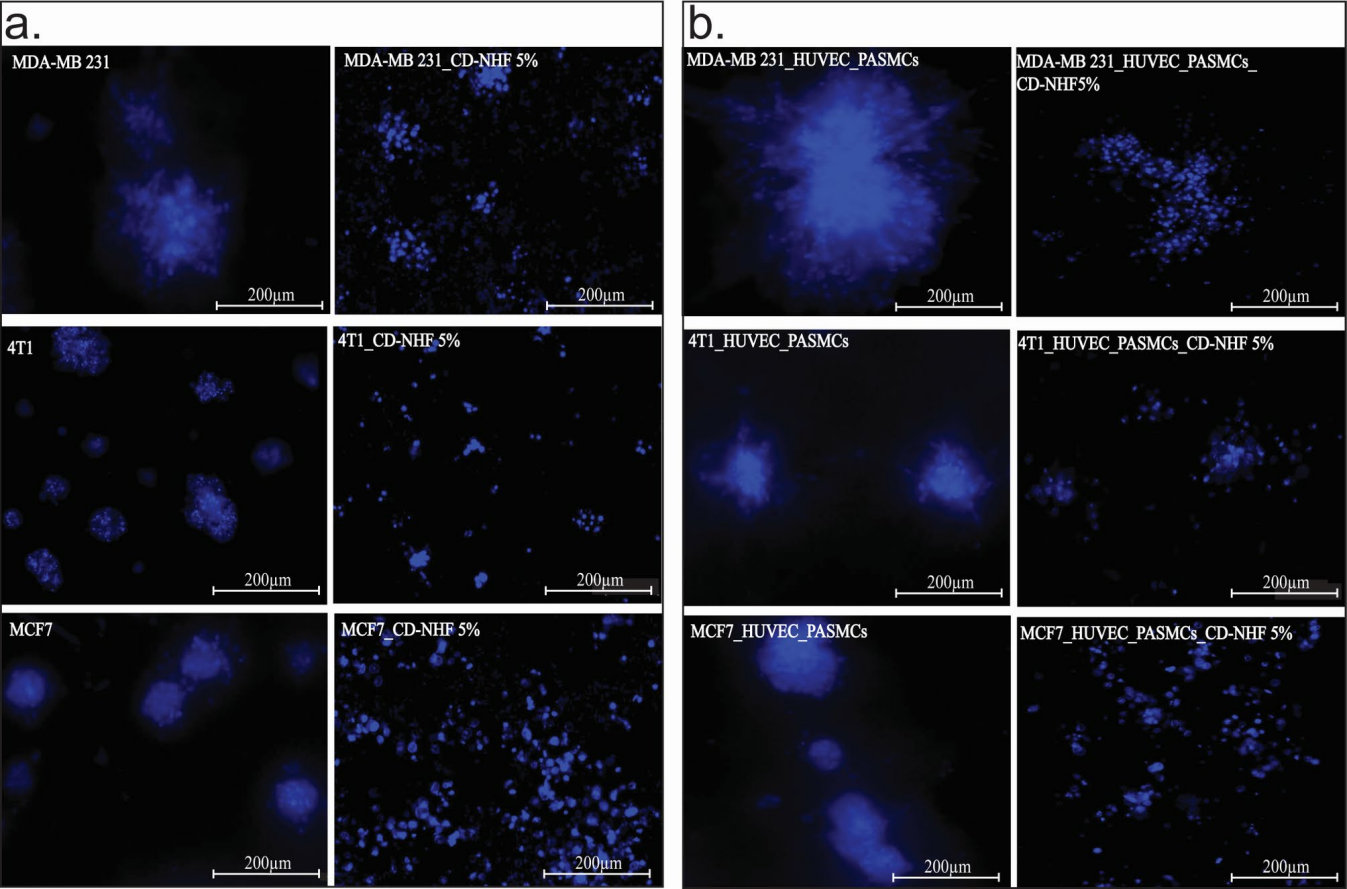
Effect of CD-NHF in 3D matrigel assay. **A** Monoculture.**a,b** MDA-MB-231 (a-untreated, b-treated); **c**,**d** 4T1 (c-untreated, d-treated); **e**,**f** MCF7 (e-untreated, f-treated); **B**. Co-culture **a**,**b** MDA-MB-231 (a-untreated, b-treated); **c**,**d** 4T1 (c-untreated, d-treated); **e**,**f** MCF7 (e-untreated, f-treated). 10 ×, N = 3 matrigels/experiment. Pictures acquired at 10 ×.

The correct Figure 2 and accompanying legend appear below.

