# Supplementary material for: NHF-derived carbon dots: prevalidation approach in breast cancer treatment
Source: Sci Rep. 2020 Jul 29;10:12662. doi: 10.1038/s41598-020-69670-z (PMC7391642; doi:10.1038/s41598-020-69670-z)

NHF-derived Carbon Dots – Prevalidation Approach in Breast Cancer Treatment

Crina Elena Tiron[1], Gabriel Luta[1,+], Mihail Butura[1,+], Florin Zugun-Eloae[1,2], Corneliu S. Stan[3], Adina Coroaba[4], Elena-Laura Ursu[4], Gabriela Dumitrita Stanciu[5], Adrian Tiron[1,*]

S1 Fig. XPS investigation

Carbon is present in highest amounts due to the graphitic core formation during the pyrolytic process. Nitrogen and oxygen are also present due to the remnant chemical groups attached to the graphitic core.

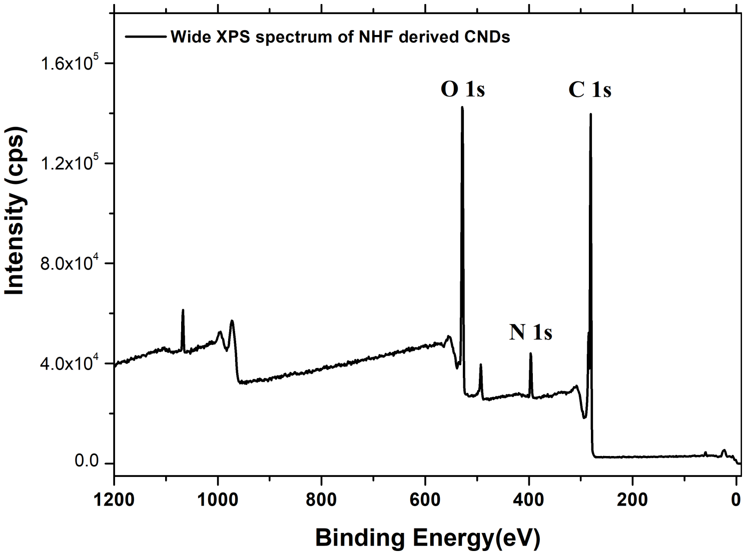

S1 Table . Total C, N, O concentrations recorded for the prepared CD-NHF.

**Element**	**Atomic concentration (%)**	**Mass concentration (%)**
O	23.52	28.85
N	4.73	5.08
C	71.75	66.07

S2 Fig. High resolution C1s, O1s and N1s spectra recording for the same CD-NHF sample.

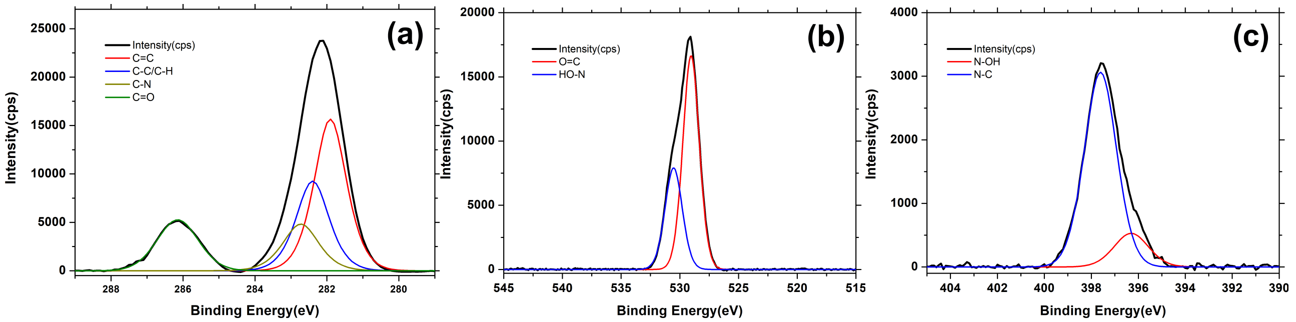

S2 Table. Relative concentrations of various atomic bondings according to C1s, O1s and N1s spectra.

**C 1s**				
Assignment	**C=C**	**C-C/C-H**	**C-N**	**C=O**
Binding energy (eV)	284.6	285	285.4	288.8
Relative concentration (%)	43.03	24.77	14.42	17.78
**O 1s**				
Assignment	**O=C**	**O-N**		
Binding energy (eV)	531.6	533.1		
Relative concentration (%)	66.26	33.74		
**N 1s**				
Assignment	**N-OH**	**N-C**		
Binding energy (eV)	398.9	400.2		
Relative concentration (%)	15.67	84.33		

The high resolution C1s spectrum revealed a preponderant presence of the C=C bondings (43.03%) which indicates the formation of the graphitic core while the presence of C-C/C-H bondings (24.77%) indicates a highly disordered configuration of the graphitic core. The presence of the surface attached functional groups are sustained by the O1s and N1s spectra with relatively high concentrations of C=O, N-C, N-O groups.

S3 Fig. Dimensional analysis

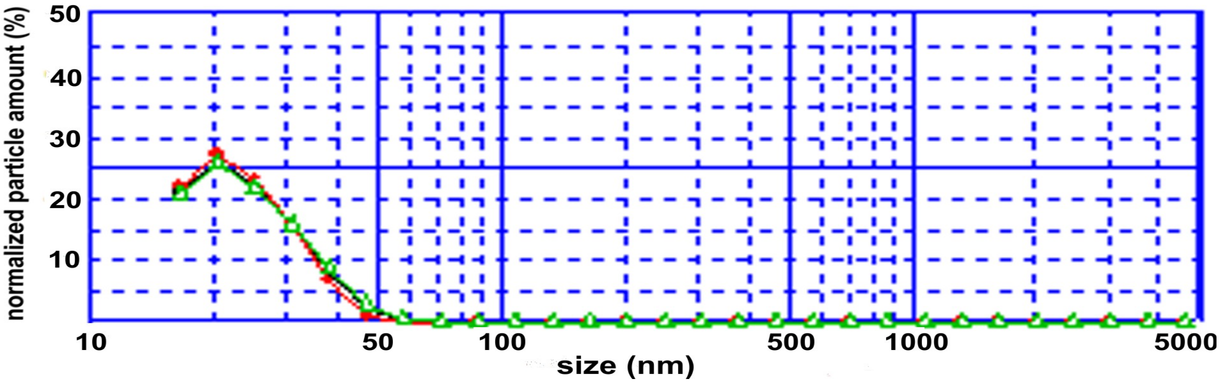

S4 Fig. AFM investigation

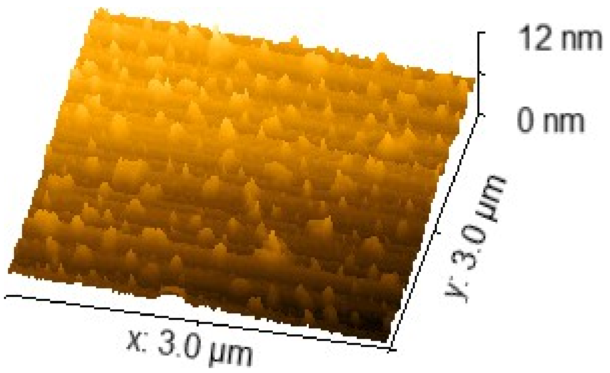

S5 Fig. Morphological aspects of cells in 3D matrigel assay just before CD-NHF treatment (5x)

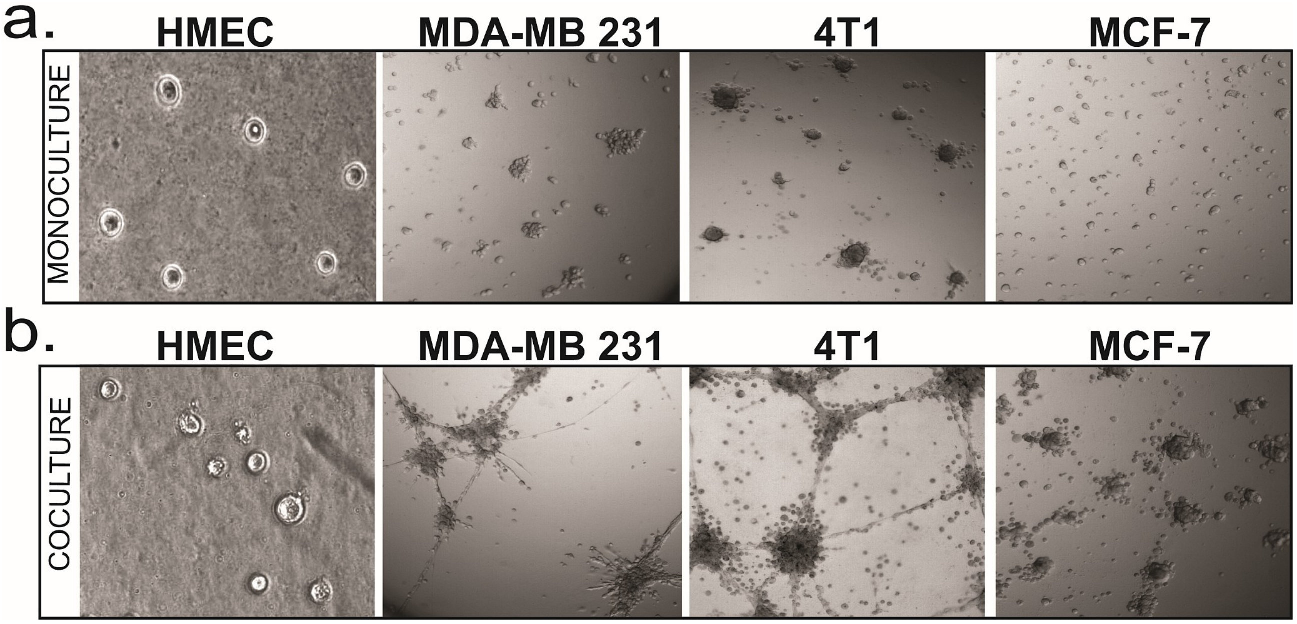

Supplement: Supplementary file 1 — Supplementary information [file 41598_2020_69670_MOESM1_ESM.pdf]
